# *Salmonellatyphi* and endocarditis: a systematic review of case reports

**DOI:** 10.3389/fmed.2024.1363899

**Published:** 2024-06-28

**Authors:** Kokab Jabeen, Sameen Bint Ali, Zainab Tufail, Sana Mustafa, Mahnoor Chaudhry, Muhammad J. Tahir, Muhammad Sohaib Asghar, Ali Ahmed

**Affiliations:** ^1^Department of Pathology, Lahore General Hospital, Lahore, Pakistan; ^2^Department of Pathology, Punjab Institute of Neurosciences (PINS), Lahore, Pakistan; ^3^Radiology, Pakistan Kidney and Liver Institute and Research Center, Lahore, Pakistan; ^4^AdventHealth, Orlando, FL, United States; ^5^Public Health, Riphah International University, Rawalpindi, Pakistan

**Keywords:** infective endocarditis, endocarditis, *Salmonella typhi*, *Salmonella*, typhoid

## Abstract

**Introduction:**

*Salmonella typhi*, a gram-negative bacterium responsible for typhoid fever, can infect the inner lining or valves of the heart and cause endocarditis. This systematic review aimed to report cases of *S. typhi*-associated endocarditis and its clinical features.

**Methods:**

This systematic review was reported as per the Preferred Reporting Items for Systematic Review and Meta-Analysis (PRISMA) checklist. Only case reports and case series of endocarditis caused by *S. typhi*, irrespective of age, gender, and demographics, were considered eligible for inclusion. To identify relevant studies, a literature search was conducted using relevant keywords on PubMed, Google Scholar, and the Cochrane Library from inception to 31 December 2023. After selecting the studies, the relevant data were extracted and pooled in terms of frequencies and percentages. A quality assessment was performed using the Joanna Briggs Institute Critical Appraisal Checklist for Case Reports.

**Results:**

This review included seven case reports, comprising 22.2% female and 77.8% male patients. The mean age of patients was 27.9 + 12.0 years. Regarding past medical history, 33.3% (3/9) of patients had a previous cardiac pathology. Fever remained the most common complaint, occurring in 88.9% of cases. Transthoracic and transesophageal echocardiography were used to diagnose all cases, with 33.3% identifying vegetation on the mitral, aortic, and tricuspid valves. Ceftriaxone, with or without gentamycin, remained the choice of antibiotic for 88.9% of cases, and all patients responded to the offered treatment.

**Conclusion:**

*S. typhi*-associated endocarditis, though rare, presents unique challenges and requires timely diagnosis. This systematic review of seven cases highlights a predominantly male population affected, with a mean age in the third decade, suggesting a higher invasiveness than other causes. The findings from this study underscore the importance of early recognition and appropriate management, primarily with antibiotic therapy. Further research with larger cohorts is crucial to refine understanding and guide policymaking for this rare but life-threatening condition.

## Introduction

*Salmonella* bacteria can be classified based on their serotype, determined by two bacterial surface antigens, O and H. They are categorized as either typhoidal (causing typhoid and paratyphoid fever) or non-typhoidal (causing gastroenteritis). To date, over 2,500 different serotypes, or serovars, have been identified. Serotypes of *Salmonella* known to cause disease in humans include both non-typhoidal strains, such as Enteritidis, Typhimurium, Newport, and Javiana, and typhoidal strains, such as Typhi (causing typhoid fever), as well as Paratyphi A, B, and C (causing paratyphoid fever). Additionally, Salmonella is further categorized into two species based on phenotype: *Salmonella enterica* and *Salmonella bongori*, with *S. enterica* further divided into six subspecies: arizonae, diarizonae, houtenae, salamae, indica, and enterica (1, 2).

*Salmonella typhi*, a gram-negative bacterium belonging to the *S. enterica* subspecies, primarily spreads via the consumption of contaminated food and water (3, 4). Globally, typhoid fever accounts for 11–21 million cases and 200,000 deaths annually (5). Owing to poor hygiene, sanitation, lack of safe drinking water, and overcrowding, the condition is more prevalent in low-to-middle-income parts of the world, whereas the history of international travel and immigration are among the significant risk factors observed in high-income regions (4, 5).

The infectious dose varies from 10,000 to 1 million organisms and is modulated by multiple factors such as geographic location and immunity status (6). Following ingestion, an incubation period of 1–2 weeks is observed, and later, the patient may present with typical step ladder fever, chills, a dull headache, nausea, malaise, anorexia, abdominal discomfort, diarrhea, or constipation. Additionally, patients may also demonstrate rose spots, a coated tongue, hepatomegaly, splenomegaly, and bradycardia (6). While most cases resolve without medical treatment or with minimal outpatient management, approximately 10–15% of patients may develop complications, with gastrointestinal bleeding being the most common. Other complications reported include intestinal perforation, cholecystitis, pneumonia, miscarriage in pregnant individuals, encephalopathy, endocarditis, and myocarditis (6). Among complications, cardiovascular involvement is rare, but cases of myocarditis, myocardial abscess, pericarditis, and aortic aneurysm have been reported (7).

Infective endocarditis (IE), a condition marked by inflammation of the endocardium, heart valves, or indwelling cardiac devices, shares an annual incidence of almost 3–10 for every 100,000 individuals (8). With a monthly mortality rate of 30%, the in-hospital mortality rate stands at 23% for patients with native valves and 29% for prosthetic valve patients (9). *Staphylococcus aureus* remains the most common causative agent, accounting for 26.6% of cases, followed by *Streptococcus viridians*, other Streptococcus groups, and Enterococci in reducing order of frequency (10). Rare cases of *S. typhi*-associated IE have also been reported in the literature (7). The reported prevalence of *Salmonella*-caused endocarditis varies from 0.01 to 2.9%, of which multiple cases result from infection with *Salmonella paratyphi*, Typhimurium, etc. (7). Owing to significant short- and long-term morbidity and mortality, appropriate treatment must be offered at the earliest. While most cases of *Salmonella*-related endocarditis are caused by non-typhoidal species, rare incidences of *S. typhi*-associated endocarditis have been reported in the literature. This may be attributable to a higher incidence of infections by non-typhoidal species (2, 11). This systematic review aimed to comprehensively evaluate the literature for clinical presentation, examination findings, investigations, management offered, and outcomes across published cases of *S. typhi*-associated endocarditis and contribute to a better understanding of these rare incidences.

## Methods

This systematic review was conducted in accordance with the Preferred Reporting Items for Systematic Review and Meta-Analysis (PRISMA) guidelines (12), and a protocol was registered at PROSPERO before the literature search (CRD42023470910).

### Search strategy

To identify all eligible studies, a literature search was conducted using PubMed, Google Scholar, and Cochrane Library from inception to 31 December 2023. The Medical Subject Heading (MeSH) browser was used to determine relevant keywords, and the search string comprised the following terms: “endocarditis,” “infective endocarditis,” “*S. typhi*,” “*S. enterica* Typhi,” “*S. typhi*,” “*S. enterica* serovar Typhi,” and “Typhoid.” To obtain comprehensive literature search results, spelling variants and synonyms were also used. Furthermore, gray literature, references of included articles, and relevant reviews were also screened to identify any missed articles.

### Inclusion and exclusion criteria

Only studies that met the predefined inclusion criteria were included (Table 1). Case reports and case series reporting endocarditis caused by *S. typhi*, irrespective of age, gender, and demographics, were deemed eligible for inclusion. Moreover, only articles published in the English language were considered. In contrast, studies reporting endocarditis caused by other *Salmonella enterica* subspecies, such as *S. typhi* murium and Paratyphi, were excluded. Similarly, cross-sectional studies, cohorts, randomized controlled trials (RCTs), narrative reviews, systematic reviews, comments, editorials, and articles published in languages other than English were not considered.

**Table 1:** Summary of included case reports.

First Author, Study Year	Patient’s characteristics	Clinical presentation	Investigations	Management	Outcome
du Plessis et al. (14)	Age: 11 y/o Sex: M Past history: Non-significant No history of valve repair or replacement	Symptoms: Abdominal pain, painful legs, and confusion for 6 days Examination: Mildly obtunded, right-sided CHF, tender 4 cm pulsatile liver, poor volume pulses, cool extremities, BP supine 72/50 mmHg, HR 100 bpm, grossly enlarged heart, parasternal heave on the right and left sternal borders, and 3/6 pansystolic murmur and 2/4 mid-diastolic murmur in the fourth ICS	Hb: 16.2 g/Dl WCC: 4000 CXR: Cardio-thoracic ratio 65% and thickened right middle lung lobe ECG: QRS axis of +135, p-pulmonale, PR interval 0.20 s, rsR pattern TTE: TR, enlarged RA, RV, Ebstein’s displacement, echo-dense vegetations on the anterior flail leaflet of the tricuspid valve Blood and stool culture = *S. typhi* positive	Digoxin, furosemide, potassium supplements, IV ceftriaxone for 3 weeks Followed by amoxicillin 2 g 6 h for 2 weeks Discharged home on oral amoxicillin for 1 more week	Patient responded to medications
Khan et al. (15)	Age:25 y/o Sex: M Past medical history = Non-significant No history of valve repair or replacement	Symptoms: Fever (102°F), generalized body ache, and palpitations for 1 week Examination: Water hammer-like bounding pulse, upper limb BP 160/69 mmHg, lower limb BP200/80 mmHg, visible carotid pulsations, and early diastolic murmur prominent in the aortic area. Some peripheral signs of aortic regurgitation were also present	Hb: 105 g/L TLC: 5600/mm3 Platelets: 2.5 × 109/L ESR: 46 mm/h Widal test: Positive Cultures: *S. typhi* positive serial blood and urine cultures (sensitive to amikacin, ofloxacin, sparfloxacin, and ceftriaxone) Echocardiography: Bicuspid aortic valves with vegetations from both cusps in the LV outflow area during diastole	Ceftriaxone 3 gm and amikacin 15 mg/kg daily for the first 2 weeks Then ceftriaxone 3 gm daily for another 2 weeks Full recovery during this period and was discharged on penicillin prophylaxis for rheumatic fever	Patient responded to medications
Khanal et al. (16)	Age: 27 y/o Sex: F Past history: RHD with MS and MR. Evaluated for breathlessness 1 month prior, where echocardiography revealed moderate MS, MR, and mild TR Drug history: Regular prophylaxis for rheumatic fever with long-acting penicillin. No history of valve repair or replacement	Symptoms: Fever, SOB, and generalized swelling for 15 days Examinations: Enlarged, firm, non-tender spleen palpable 4 cm below the costal margin. Chest examination was normal	Hb: 10 g% Urine Analysis: RBCs positive Blood culture: *S. typhi* (sensitive to amikacin, chloramphenicol, ciprofloxacin, and gentamicin) TTE: Large vegetation attached to mitral leaflets in addition to her valvular defects	Patient was treated with ciprofloxacin and gentamicin to which she responded	Patient responded to medications
Ozer et al. (17)	Age: 27 y/o Sex: F Past medical history: Rheumatic valve disease 34 weeks pregnant No history of valve repair or replacement	Symptoms: Fever, anorexia, and general weakness for 15 days Examination: HR 110/min, BP 105/55 mm Hg, diastolic murmur involving the apical region and left sternal edge	ESR: 100 mm/h CRP: 151 mg/L WCC: 7630/Ul Hb: 12.9 g/dL Platelets: 143,000/ul TTE: MS, AR with shortened, thickened valve TEE: 10 × 6 mm, partially mobile vegetation attached to the cusp of the aortic valve Blood cultures: *S. typhi* positive	Empirical: IV penicillin and gentamycin therapy After culture: 4 g Ceftriaxone twice daily. Delivery was performed via CS following a course of steroids and antibiotic treatment continued for 6 weeks until the patient recovered	Patient responded to medications
Arif Khan et al. (18)	Age: 21 y/o Sex: M Past medical history: Mitral valve repair for MR	Symptoms: Fever and diarrhea for 1 month, and SOB for 3 days Examination: Fever (38°C), HR 80/min, regular pulse, BP 100/60 mmHg, mild systolic and a diastolic murmur barely audible, and scattered rhonchi in lung fields	Chest radiograph: Normal ECG: LVH, non-specific ST-T changes in lateral chest leads TLC: 14,000/mL *Salmonella* O and H antigens antibodies: Positive Blood culture: Positive for *S. typhi* (sensitive to ceftriaxone and cefixime) Echocardiography: Small echogenic mitral masses with mild-to-moderate MR and mild AR	Empirical antibiotics = IV Penicillin G and gentamycin After culture = IV ceftriaxone 2 gm twice daily and gentamycin 60 mg, 8 hourly for 2 weeks. Followed by ceftriaxone continued for another 2 weeks The patient improved after starting ceftriaxone	Patient responded to medications
Palangasinghe et al. (19)	Age: 55 y/o Sex: M Past history: Type II DM. Driver by occupation with poor food Hygiene No history of valve repair or replacement	Symptoms: High-grade fever, frontal headache, nausea, anorexia for 2 weeks Examination: Fever (102 F), BP 140/90 mmHg, no cardiac murmurs, soft splenomegaly with spleen palpable 3 cm from the left costal margin	HB: 10.5 g/ dL WCC: 5600/ mm3 Platelets: 165000/mm^3^ ESR: 102 CRP: 65.4 mg/L Urine analysis: 10–15 RBC/HPF Blood cultures: *S. typhi* (sensitive to ceftriaxone) TTA and TEE: 5 × 4 mm vegetation attached to the non-coronary cusp of a tricuspid aortic valve	IV ceftriaxone 2 g twice daily and gentamicin 60 mg twice daily for 2 weeks. Then, ceftriaxone 2 g twice daily for another 2 weeks TEE showed complete resolution of vegetation in the third week	Patient responded to medications
Robson et al. (20)	Age: 20 y/o Sex: M Race: Filipino Past medical history: Non-significant Recent 3-week trip to Philippine No history of valve repair or replacement	Disease Course: Mild headache, fever, and diarrhea 2 weeks after returning but did not respond to medicines, leading to ER visit after 1 week with fever, hypotension, tachycardia, soft abdomen, and mild tenderness in RUQ. Blood cultures were positive for *S. enterica* serovar Typhi (sensitive to ampicillin, ceftriaxone, cotrimoxazole, azithromycin, and ciprofloxacin). Treated with ceftriaxone for 8 days and 7 days oral course of ciprofloxacin, and condition improved ER visit after 6 weeks: Symptoms: Fever, mild headache, and lethargy for the last 5 days Examination: Dual heart sounds without any murmurs or any peripheral stigmata of IE On day 3, the patient developed a short systolic murmur in the tricuspid area	Hb: 123 g/L WCC: 2.3 × 109/L Platelets: 127 × 109/L Cr: 92 μmol/L CRP: 110 mg/L ALT: 117 U/L AST 194 U/L GGT 30 U/L ALP 67 U/L Bilirubin 10 μmol/L Blood cultures, stool samples, and PCR were positive for *S. typhi* Both TTE and TEE revealed a 4 mm small, mobile vegetation on the anterior leaflet of the mitral valve	Treated with IV ceftriaxone 2 g/day for 6 weeks followed by oral ciprofloxacin 500 mg twice daily for another 6 weeks The blood cultures cleared within a day and symptoms resolved within 7 days	Patient responded to medications
Kadappu and Kainthaje (21)	Sex: M Age: 40 y/o No history of valve repair or replacement	Onset: 4–5 episodes of watery diarrhea Presentation: Fever and joint pain for 3 weeks Examination: Dual heart sounds without any murmur or pericardial friction rub	Hb: 92 gm/L WBC: 12×109 /L Bilirubin: 36 IU AST: 79 IU ALT: 60 IU ALP: 370 IU Doppler study revealed vegetation in the tricuspid valve (2·5 cm × 2 cm) with mild tricuspid regurgitation Day 5: *S. typhi* isolated on blood culture (sensitive to ceftriaxone and gentamicin)	Initially: Ciprofloxacin 200 mg twice daily After culture: Ceftriaxone 2 gm twice daily and gentamicin 60 mg thrice daily for 14 days. Ceftriaxone continued for 2 more weeks	Patient responded to medications
Zahoor et al. (22)	Sex: M Age: 25 y/o Working in a tuberculosis center No history of valve repair or replacement	Presentation: Cough for 45 days (white sputum, which changed to red), fever (associated with rigors, chills, and night sweats) for 30 days, shortness of breath for 30 days, and hemoptysis for 3 days Examination revealed dullness to percussion in the right mid-zone lungs, and auscultation of the bilateral upper zone revealed wheezes, bilateral mid-zone crackles, palpable spleen tip, early diastolic murmur in the aortic (A2) area	Hb: 10.9 g/Dl WBC: 20.4 × 103/uL Sputum AFB, Gene Xpert: negative Echocardiogram: multiple aortic valve vegetation, severe aortic regurgitation (non-co-optative aortic valve), and moderate mitral regurgitation Chest HRCT: bilateral dense multi-lobular shadowing, ground-glass opacification, interlobular and intra-lobular septal thickening, and multiple sub-centimeter mediastinal lymph nodes Blood culture growth of *S. typhi* (sensitive to imipenem, meropenem, ceftriaxone, and cefixime)	Ceftriaxone (2 gm BID), meropenem (2 gm BID) for 2 weeks	Patient responded to medications

y/o, years old; M, male; F, female; ER, emergency; RUQ, right upper quadrant; GR, heart rate; BP, blood pressure; Hb, heamoglobin; WCC, white cell count; TLC, total leukocyte count; Cr, creatinine; CRP, C-reactive protein; ALT, alanine transaminase; ALT, aspartate transaminase; GGT, gamma-glutamyl transferase; TTE, transthoracic echocardiography; TTE, transesophageal echocardiography; ALP, alkaline phosphatase; IV, intravenous; LV, left ventricle; RV, right ventricle; RA, right atrium; LVH, left ventricular hypertrophy; ECG, electrocardiography; MR, mitral regurgitation; AR, atrial regurgitation; TR, tricuspid regurgitation, MS, mitral stenosis; AS, aortic stenosis; RHD, rheumatic heart disease; CHF, congestive heart failure; CS, cesarean section; SOB, shortness of breath; CXR, chest X-ray; DM, diabetes mellitus; RBC, red blood cells; hpf, high power field; PCR, polymerase chain reaction; dL, deciliter; U, units; mg, milligram; L, liter; μmol, micro-mole.

### Study screening and data extraction

Following a thorough literature search, two authors (KJ and SBA) independently screened all the search results via title and abstract. Recruited articles were then examined at full length to determine adherence to the inclusion criteria. Only articles meeting the above-mentioned inclusion criteria were included.

After the study selection process, two independent authors (KJ and SBA) extracted the following data from all included case reports: first author’s last name, year of publication, patient age, gender, past medical history, any other relevant baseline features, the clinical course of the disease, presenting complaints, examination findings, investigations ordered, drugs offered, management course, and outcome into an excel spreadsheet.

### Data synthesis and quality assessment

Excluding age, which was expressed as the mean age of included participants, all other parameters were reported as frequencies and percentages. To assess the quality of each included study, the Joanna Briggs Institute Critical Appraisal Checklist for Case Reports (13) was used, which scrutinizes each included study based on the reported patient’s demographics, clinical history, presentation, examination findings, diagnostic investigations, interventions offered, post-intervention impact, adverse events reported, and take-home messages.

## Results

### Literature search

**Figure 1:**
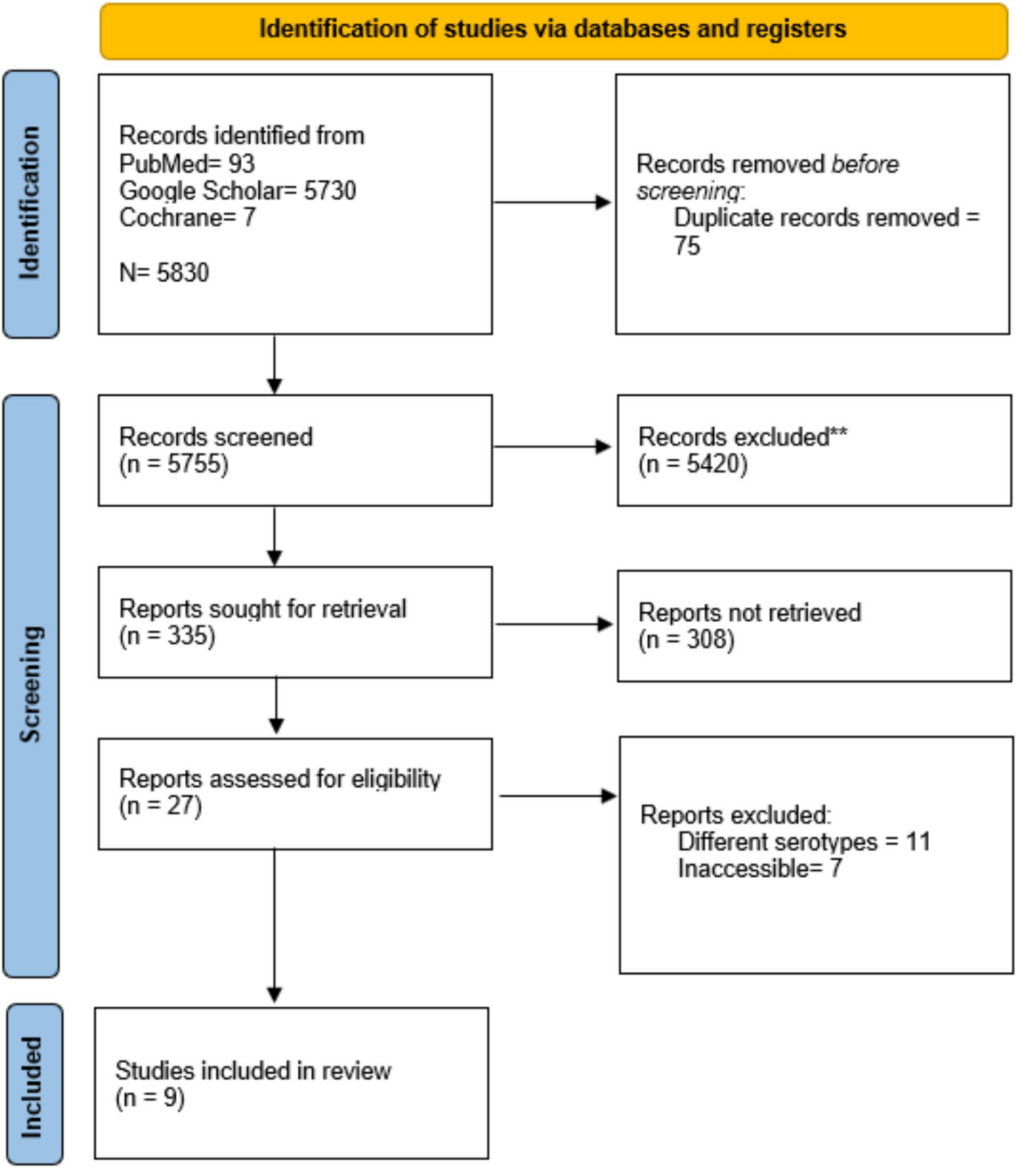
Preferred reporting items for systematic review and meta-analysis (PRISMA) flowchart.

Our initial literature search revealed 5,830 articles: 93 records from PubMed, 5,730 from Google Scholar, and 7 articles from the Cochrane Library. Following duplicate removal and screening via title and abstract, 27 case reports were considered for full-length review. Ultimately, only nine studies met the predefined inclusion criteria and were included in this systematic review (14–22). The results of the literature search are summarized in Figure 1.

### General characteristics of included studies

The included case reports comprised data from nine patients, and their baseline characteristics, clinical presentation, laboratory investigations, and management offered are summarized in Table 1. Of the included population, 22.2% (2/9) (16, 17) were female patients, and the remaining 77.8% (7/9) (14, 15, 18–22) were male patients. The participants’ ages ranged from 11 to 55 years, with a mean age of 27.9 + 12.0 years. Regarding past medical history, 33.3% (3/9) (16–18) of patients had a previous cardiac pathology, with 22.2% (2/9) (16, 17) being diagnosed with rheumatic heart disease (RHD). In contrast, 55.6% (5/9) (14, 15, 20–22) had a non-significant past medical history and 11.1% (1/9) (19) had type II diabetes mellitus.

### Clinical features across included cases

At the time of emergency presentation, fever was the most common complaint, present in 88.9% (8/9) (15–22) of patients. Gastrointestinal complaints were present in only 33.3% (3/9) (14, 18, 21) of individuals, with 22.2% (2/9) (18, 21) having diarrhea and the remaining 11.1% having abdominal pain (14). Cardiac symptoms were present in 44.4% (4/9) (15, 16, 18, 22) of patients: shortness of breath in 33.3% (3/9) (16, 18, 22) and palpitations in 11.1% (1/9) (15) of participants. Other presenting complaints included headaches (22.2%) (19, 20), bone and joint pain (2.2%) (14, 21), generalized pain (11.1%) (15), confusion (11.1%) (14), weakness and lethargy (22.2%) (17, 20), generalized swelling (11.1%) (16), anorexia (22.2%) (17, 19), cough (11.1%) (22), hemoptysis (11.1%) (22), and nausea (11.1%) (19).

Upon examination, murmurs were the most common finding, being present in 55.6% (5/9) of cases (14, 15, 17, 18, 22). Blood pressure and pulse abnormalities were found in 33.3% (3/9) of patients (14, 15, 17). Similarly, splenomegaly was determined in 33.3% (3/9) (16, 19, 22) of cases. Other findings reported include peripheral signs of aortic regurgitation (11.1%) (15), hepatomegaly (11.1%) (14), gross cardiomegaly (11.1%) (14), right-sided congestive heart failure (11.1%) (14), and dual heart sounds (22.2%) (20, 21). The clinical presentation and examination findings are summarized in Table 1.

### Laboratory investigations among included cases

Transthoracic and transesophageal echocardiography remained the mainstay of diagnosis in all cases, with identified vegetation involving the mitral (16, 18, 20), aortic (15, 17, 22), and tricuspid valves (14, 19, 21), each in 33.3% (3/9) of individuals. Other findings identified included right atrial and ventricular enlargement, bicuspid aortic valve, left ventricular hypertrophy, mitral stenosis, mitral regurgitation, and aortic regurgitation. A blood culture was ordered in all nine cases, and each tested positive for *S. typhi*. Other investigations ordered include a complete blood count (CBC), erythrocyte sedimentation rate (ESR), urine analysis, electrocardiography (ECG), chest radiograph, liver function test (LFT), and Widal test.

### *Salmonella typhi* endocarditis management across included cases

Following antibiotic sensitivity analysis, ceftriaxone remained the choice of antibiotic for 88.9% (8/9) (14, 15, 17–22) of cases. Ceftriaxone alone was initially given to 33.3% (3/9) of individuals (14, 17, 20), followed by ceftriaxone and gentamycin in 33.3% (3/9) (18, 19, 21), ceftriaxone and amikacin in 11.1% (1/7) (15), and ceftriaxone and meropenem in 11.1% (22) of individuals. The duration of treatment varied from 4 weeks to 12 weeks, and all nine patients responded to the offered intervention and recovered. No mortality was observed across any of the included studies.

### Quality appraisal

Our quality appraisal using the JBI appraisal tool revealed an overall low risk of bias across included studies except for reporting of adverse or unanticipated events, which were marked unclear for all studies due to a lack of clarity on whether any adverse event occurred. Additionally, the take-home message was adequately specified in only 66.7% (6/9) of studies (15, 18–22), being unclear or not mentioned in the remaining 3. Other than this, the patient’s demographics, history, and current presentation were discussed across all included studies. The results of the quality assessment are summarized in Table 2.

**Table 2:** Results of quality assessment.

Author, Year	Were the patient’s demographic characteristics clearly described?	Was the patient’s history clearly described and presented as a timeline?	Was the current clinical condition of the patient on presentation clearly described?	Were diagnostic tests or assessment methods and the results clearly described?	Was the intervention(s) or treatment procedure(s) clearly described?	Was the post-intervention clinical condition clearly described?	Were adverse events (harms) or unanticipated events identified and described?	Does the case report provide takeaway lessons?
du Plessis et al. (14)	Yes	Yes	Yes	Yes	Yes	Yes	Unclear	No
Khan et al. (15)	Yes	Yes	Yes	Yes	Yes	Yes	Unclear	Yes
Khanal et al. (16)	Yes	Yes	Yes	Unclear	No	Yes	Unclear	Unclear
Ozer et al. (17)	Yes	Yes	Yes	Yes	Yes	Yes	Unclear	Unclear
Arif Khan et al. (18)	Yes	Yes	Yes	Yes	Yes	Yes	Unclear	Yes
Palangasinghe et al. (19)	Yes	Yes	Yes	Yes	Yes	Yes	Unclear	Yes
Robson et al. (20)	Yes	Yes	Yes	Yes	Yes	Yes	Unclear	Yes
Kadappu and Kainthaje (21)	Yes	Yes	Yes	Yes	Yes	Yes	Unclear	Yes
Zahoor et al. (22)	Yes	Yes	Yes	Yes	Yes	Yes	Unclear	Yes

## Discussion

This systematic review comprehensively assesses the literature for case reports and case series reporting rare incidences of endocarditis caused by *S. typhi* and includes data from nine published cases. The condition demonstrated a male predominance, majorly affecting individuals in their 20s. Although the clinical presentation varied among studies, fever, lethargy, and cardiac and gastrointestinal symptoms were frequently reported. In terms of valve involvement, mitral, aortic, and tricuspid valves were equally involved. Antimicrobial therapy, particularly ceftriaxone with or without gentamycin, remained the mainstay of management, and all patients responded positively to the offered treatment.

The mean age of presentation and gender distribution vary across different studies. According to Cheng et al. (7), *Salmonella* endocarditis showed female predominance, with a mean age of presentation estimated at 40 years. Contrary to the above finding, our systematic review revealed a male preponderance and presentation in the third decade. This difference may be attributable to the diverse studies covered in the review by Cheng et al., which included a majority of cases caused by non-typhoid *Salmonella* subspecies.

While RHD is the most common risk factor for IE among adults, accounting for 55% of affected individuals and 44.7% of affected children having a positive history of congenital cardiac pathologies (23), our findings indicate a lower incidence, with any prior cardiac pathology present in less than half of participants and RHD in less than a third. Similar findings were reported by Cheng et al., where a younger mean age and a relative absence of pre-existing cardiac diseases were found in *S. typhi* and Paratyphi-related cases, indicating a higher invasiveness compared to non-typhoid *Salmonella*-associated cases (7).

In accordance with our results, a recent prospective study by Habib et al. also reported fever as the most common symptom in patients with IE (24). Moreover, preceding abdominal complaints were observed in one-third of the cases reported. A similar proportion of gastrointestinal symptoms was reported in the review by Cheng et al. Other clinical features observed in the included cases, such as body aches, murmurs, splenomegaly, and shortness of breath, have also been commonly reported in the literature (20, 21).

Furthermore, in accordance with the hospital guidelines, echocardiography remained the mainstay of diagnosis across all nine included studies (22, 23). The blood cultures were positive for the causative organisms in all nine cases, as compared to 79% in a recent prospective cohort (24). Generally, IE more commonly affects left-sided valves, with the mitral valve being involved in most cases (52.2%), followed by an aortic valve (34.8%) (25). Similarly, in a review including *Salmonella*-associated endocarditis cases, the mitral valve was most frequently involved, affecting approximately 33.3% of patients (7). In congruence with the above findings, our review also found the mitral valve to be most affected, but the aortic and tricuspid valves also demonstrated similar involvement.

In accordance with the guidelines published in the Journal of American Medical Association, antibiotic therapy remained the mainstay of management, and the choice of drug was guided primarily by the organism’s sensitivity (26). However, in none of the included cases was a surgical approach offered, despite literature reports of lower mortality rates in patients offered combined medical and surgical interventions (15%) compared to those treated with medical therapy only (27.2%) (5, 27). Owing to better diagnostic techniques and prompt management, the overall prognosis of *Salmonella* endocarditis has improved over the last three decades, with the mortality rate reducing from 69.0 to 13.3% (5, 27, 28).

Excluding endocarditis, other cardiovascular complications such as *Salmonella* pericarditis, mycotic aneurysms, and infection of the arteriovenous fistula have also been reported in the literature (29, 30, 31). Hence, timely identification of high-risk patients and prompt management are necessary to avoid any preventable mortality.

## Limitations

This study has certain limitations. First, it encompasses a limited number of studies. Second, this systematic review exclusively encompasses case reports featuring small sample sizes and individual patient-level data. Consequently, extrapolating the findings of this study to a broader population becomes difficult. Finally, the absence of control subjects in the studies included may introduce potential biases in the study’s results. Hence, large group studies must be conducted to determine differences among risk factors, clinical presentation, desired investigations, and management for *S. typhi* endocarditis relative to other causative agents. Finally, in our systematic review of case reports, we recognize the potential for publication bias, wherein positive or exceptional cases may be preferentially published, possibly influencing the overall findings.

## Conclusion

This systematic review scrutinized seven case reports of *S. typhi*-associated endocarditis, elaborating on unique clinical presentations, diagnostic investigations, and management approaches. Notably, a predominantly male and relatively younger population was affected compared to endocarditis of other origins, indicating a higher invasiveness. Fever remained the most common presenting feature, with abdominal pain, headache, body aches, palpitations, splenomegaly, murmurs, and others also reported. Echocardiography and positive blood cultures were pivotal in the diagnosis, with the mitral valve frequently affected. Antibiotic therapy remained the primary treatment, yielding promising outcomes, although surgical interventions were notably absent.

## Data availability statement

The original contributions presented in the study are included in the article/supplementary material, further inquiries can be directed to the corresponding author.

## Author contributions

KJ: Conceptualization, Data curation, Writing – original draft, Writing – review & editing. SB: Formal analysis, Writing – original draft, Writing – review & editing. ZT: Investigation, Software, Writing – original draft, Writing – review & editing. SM: Methodology, Resources, Writing – original draft, Writing – review & editing. MC: Data curation, Resources, Writing – original draft, Writing – review & editing. MT: Project administration, Supervision, Writing – original draft, Writing – review & editing. MA: Project administration, Supervision, Writing – original draft, Writing – review & editing. AA: Validation, Visualization, Writing – original draft, Writing – review & editing.
